# Intra and Extracellular Journey of the Phytohormone Salicylic Acid

**DOI:** 10.3389/fpls.2019.00423

**Published:** 2019-04-16

**Authors:** Israel Maruri-López, Norma Yaniri Aviles-Baltazar, Antony Buchala, Mario Serrano

**Affiliations:** ^1^Centro de Ciencias Genómicas, Universidad Nacional Autónoma de México, Cuernavaca, Mexico; ^2^Instituto de Investigación en Ciencias Básicas y Aplicadas, Universidad Autónoma del Estado de Morelos, Cuernavaca, Mexico; ^3^Department of Biology, University of Fribourg, Fribourg, Switzerland

**Keywords:** salicylic acid, transport, phytohormone, defense response, systemic acquired resistance, plant-microbe interactions

## Abstract

Salicylic acid (SA) is a plant hormone that has been described to play an essential role in the activation and regulation of multiple responses to biotic and to abiotic stresses. In particular, during plant-microbe interactions, as part of the defense mechanisms, SA is initially accumulated at the local infected tissue and then spread all over the plant to induce systemic acquired resistance at non-infected distal parts of the plant. SA can be produced by either the phenylalanine or isochorismate biosynthetic pathways. The first, takes place in the cytosol, while the second occurs in the chloroplasts. Once synthesized, free SA levels are regulated by a number of chemical modifications that produce inactive forms, including glycosylation, methylation and hydroxylation to dihydroxybenzoic acids. Glycosylated SA is stored in the vacuole, until required to activate SA-triggered responses. All this information suggests that SA levels are under a strict control, including its intra and extracellular movement that should be coordinated by the action of transporters. However, our knowledge on this matter is still very limited. In this review, we describe the most significant efforts made to date to identify the molecular mechanisms involved in SA transport throughout the plant. Additionally, we propose new alternatives that might help to understand the journey of this important phytohormone in the future.

## Discovery of Salicylic Acid

Salicylic acid (SA) belongs to a group of molecules collectively named salicylates, which are phenolic compounds synthesized by plants, that possess an aromatic ring and a hydroxyl group. Even before salicylates were chemically identified, for thousands of years, humans used them as pain killers (Klessig et al., 2018). The first known evidence of this use was discovered on the dental plaque from Neanderthals fossils from El Sidrón cave (Weyrich et al., 2017). These fossils contained remains of poplar bark, suggesting that the individuals chewed the plant to relieve the pain of dental abscesses. Use of plants containing salicylates as analgesics, can be found across many ancient cultures from Europe, Asia and America (Vlot et al., 2009). Nevertheless, it was not until 1828 that salicin was purified from willow bark by Johann A. Buchner. Subsequently around 1838, salicin was separated into a sugar and an aromatic compound that could be converted into an acid, named salicylic acid (2-hydroxybenzoic acid), after the Latin name of the white willow tree *Salix alba* by Raffaele Piria (Vlot et al., 2009; Kumar, 2014). Free SA is poorly soluble in water and very soluble in polar organic solvents, with a pH value of a saturated aqueous solution of 2.4. SA fluoresces at 412 nm when excited at 301 nm and this property has been used to detect it in several plants (Raskin, 1992).

In 1859, Hermann Kolbe and coworkers synthesized SA, leading to an increase in its consumption due to easy availability and decreased cost. Then, years later, in order to avoid side effects induced by SA consumption (irritation and bleeding in the stomach), Felix Hoffmann reported that acetylsalicylic acid, caused less damage to the digestive system, which finally ended in the product that is nowadays used worldwide – aspirin (Vlot et al., 2009; Klessig et al., 2018). Even though the effects and benefits of aspirin in humans to treat fever, pain or swelling and to reduce the risk of heart attack, stroke and certain cancers, have been well described and studied (Klessig et al., 2018), its role as secondary metabolite in plant biology was only characterized in the late 20th century.

## The Plant Hormone SA

Plant hormones have been described to play essential biological roles regulating plant growth, development, reproduction and survival; and many of these mechanisms are regulated by cross-communication and signal-transduction pathways, within which plant hormones fulfill central roles (Verhage et al., 2010; De Vleesschauwer et al., 2013).

For many years, SA was considered just one of the thousands of phenolic compounds produced by the plants, another secondary metabolite with a relatively unimportant biological function (Raskin, 1992; Métraux and Raskin, 1993). However, in 1974, SA was described for the first time as a mobile signaling molecule localized in the phloem, that can induce flowering of *Xanthium strumarium* and *Lemna gibba*, suggesting a role as a plant hormone (Cleland, 1974; Cleland and Ajami, 1974). Nevertheless, the evidence that SA was a phytohormone came from the description of its role during the thermogenesis in voodoo lily (*Sauromatum guttatum*) (Raskin et al., 1989). The authors identified a 100-fold increase of endogenous SA during this event that can be also specifically stimulated by exogenous SA or its derivatives, but not by other structurally related compounds. After this initial characterization, multiple reports studied the role of SA as a phytohormone, including its involvement in the resistance and tolerance to many abiotic stresses, including ozone, UV radiation, paraquat, heat, cold, metal, and salinity/osmotic stresses (Horváth et al., 2007; Yuan and Lin, 2008; Rivas-San Vicente and Plasencia, 2011; Dempsey and Klessig, 2017). In addition, there is evidence that application of SA affects multiple aspects of plant growth and development, including seed germination, vegetative growth, flowering, fruit yield, senescence, stomatal closure, thermogenesis, photosynthesis, respiration, changes in the alternative respiratory pathway, glycolysis and the Krebs cycle (Khan et al., 2015; Dempsey and Klessig, 2017; Klessig et al., 2018). Nevertheless, one of the better- characterized SA-induced responses is that involving plant-microbe interactions, described below.

## SA, An Essential Regulator of Plant-Microbe Interactions

Plants are constantly interacting with millions of microorganisms, including bacteria, yeast, fungi, and viruses (Lindow and Brandl, 2003; Baldwin et al., 2017). In response, an intimate dialogue is established between plants and microbes, which modifies the growth and development of the pathogens and the induction of the plant defense responses (Aragón et al., 2017). The first line of defense is grouped into the innate immunity, which can be divided into pathogen-associated molecular pattern-triggered immunity (PTI) and effector triggered immunity (ETI). During PTI, accumulation of reactive oxygen species (ROS), mitogen activated protein kinase (MAPK)-dependent signaling cascades and transcriptional reprograming are induced, while during ETI, induction of the above PTI responses is stronger and/or more rapid, which is usually accompanied by programmed cell death or hypersensitive response at the infection site (Boller and Felix, 2009; Zipfel, 2014; Conrath et al., 2015). The effect of ETI and PTI can block the infection of unadapted pathogens, both at the local infected tissue and systemically in uninfected leaves (Craig et al., 2009). After these early events, secondary or late defense responses are triggered, including the activation of hormone-induced signaling pathways. The main hormones involved during the innate immunity are SA, jasmonic acid, and ethylene (Yang et al., 2013). However, abscisic acid, gibberellins, auxins, cytokinins, and brassinosteroids can also function as modulators of the plant immune signaling networks (Pieterse et al., 2009, 2012).

The role of SA during plant-microbe interactions, was recorded for the first time in tobacco and cucumber plants in 1990. Plants with an increased resistance to tobacco mosaic virus (TMV) showed a strong accumulation of SA, while with TMV-susceptible plants, SA levels were significantly reduced (Malamy et al., 1990). Multiple reports have been published showing the effect of SA regulating the interactions between plants and microbes and recent reviews have made detailed description of this regulation (Dempsey and Klessig, 2017; Klessig et al., 2018). Here, we describe some of the most relevant findings.

**FIGURE 1:**
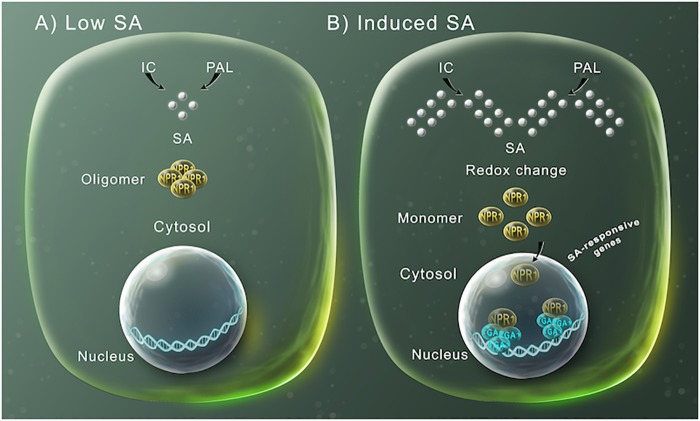
SA-mediated gene expression regulation for plant defense. **(A)** SA (silver spheres) is synthesized by either the phenylalanine ammonia-lyase (PAL) or the isochorismate (IC) pathways. At low SA levels, NPR1 occurs in the cytosol as an oligomer bound by disulfide bridges. **(B)** At high SA intracellular concentration, the increase in SA modifies the cellular reduction potential, which leads to NPR1 structure changes to monomers through the reduction of the intermolecular bridges by a change in redox. This allows NPR1 to enter the nucleus, where it binds to specific TGA transcriptions factors inducing the expression of SA-induced defensive response genes.

After the initial observation with tobacco and cucumber plants, several studies found that tobacco and *Arabidopsis thaliana* plants transformed with the bacterial gene *nahG*, encoding a bacterial salicylate hydroxylase that degrades SA to catechol, showed enhanced susceptibility to pathogens (Gaffney et al., 1993; Delaney et al., 1994; Lawton et al., 1995). Through genetic and biochemical analysis, the SA-dependent signaling pathway has been characterized during the past few years (Figure 1). Recently, the protein NPR1 was described as the receptor of SA (Wu et al., 2012). Uninfected plants, with low levels of SA, NPR1 forms an oligomeric complex localized in the cytosol (Figure 1A), modulating the cross talk between SA and jasmonic acid, but not inducing SA-dependent defense genes (Spoel et al., 2003). However, upon pathogen infection or treatment with exogenous SA, a change in the cellular redox system occurs, that has been associated with the SA-triggered suppression of JA responses (Holuigue et al., 2016), which induces NPR1-complex dissociation to monomers, by the reduction of disulfide links (Figure 1B) (Tada et al., 2008). These monomeric forms can then be translocated into the nucleus, where they interact with basic leucine zipper (bZIP) TGA-type transcription factors, inducing the modification of the transcriptome, including the transcriptional activation of the defense-related genes, such as *PR-1* (Figure 1B) (Fu and Dong, 2013; Birkenbihl et al., 2017).

Additionally, NPR1 activity is regulated by proteasome-mediated degradation. This process is carried out by the NPR1 paralogues, NPR3 and NPR4, which are adaptors for the Cullin 3 ubiquitin E3 ligase and mediates the NPR1 degradation in a SA-dependent manner. In uninfected cells, when SA levels are low, NPR4 is proposed to maintain low NPR1 levels. However, after infection, when SA levels increase, NPR4-NPR1 interaction is disrupted, allowing the accumulation of NPR1. Additionally, when the level of SA is extremely high, NPR3 binds NPR1 leading to NPR1 degradation (Spoel et al., 2009; Fu et al., 2012; Dempsey and Klessig, 2017). NPR1 turnover ensures a correct defense activation and is required for full induction of target genes and the establishment of SA-induced responses (Spoel et al., 2009). However, until now only the ubiquitin ligase adapter function has been attributed to NPR3 and NPR4, but they also might be involved in transcriptional regulation of SA-induced defense genes. *Arabidopsis thaliana* plants harboring NPR3 and NPR4 mutant versions, unable to bind SA, showed constitutive repression of SA-induced immune responses and enhanced SA-induced defense gene expression, respectively (Ding, 2018).

After these initial SA-triggered responses in the local infected/treated tissue, plants can induce a long-lasting and broad-spectrum defense syndrome called systemic acquired resistance (SAR), which includes the accumulation of PR proteins and the induction of further biosynthesis of SA (Mishina and Zeier, 2007). SAR promotes a priming of defense mechanisms which is a faster and stronger response to a secondary infection inflicted by virus, bacterial and fungal pathogens (Vlot et al., 2009; Conrath et al., 2015). The chemical derivative of SA (MeSA) was initially described as the mobile signal responsible for inducing SAR (Rasmussen et al., 1991; Park et al., 2007; Vlot et al., 2009). However, while several reports suggest that SA is an important molecule to induce SAR, it is not the only one (Smith et al., 1991; Vernooij et al., 1994). Later work has described that together with SA, other compounds participate as inducers of SAR including: dicarboxylic acid azelaic acid (AzA), glycerol-3-phosphate (G3P), dehydroabietinal (DA), and pipecolic acid (Pip) [reviewed by Singh et al. (2017)]. Nevertheless, these results show the important role of SA during the interactions between plants and pathogens.

## SA Biosynthesis and Storage Are Compartmentalized

**FIGURE 2:**
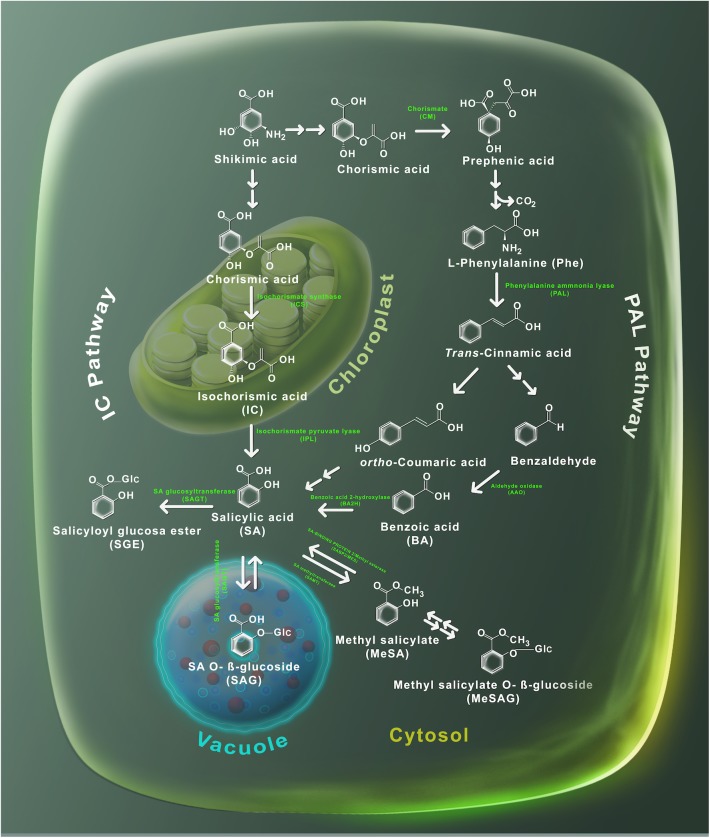
Metabolism of SA. Plants utilize two pathways to produce SA, the phenylalanine ammonia-lyase (PAL) and the isochorismate (IC). Shikimic acid serves as precursor in both routes. The PAL route is carried out in the cytosol, where shikimic acid is transformed into chorismic acid and then into phenylalanine by the action of the chorismic mutase enzyme. Later, the PAL enzyme uses phenylalanine to produce *trans*-cinnamic acid which in turn is converted into both *ortho*-coumaric acid and benzaldehyde. Subsequently, benzaldehyde is converted to benzoic (BA) acid by the aldehyde oxidase (AAO) enzyme. BA is transformed to SA through a reaction catalyzed by the benzoic acid hydroxylase (BA2H) enzyme. The IC pathway takes place at the chloroplast – SA is synthetized by the isochorismate synthase to generate isochorismate, which is later transformed into SA through the action of the isochorismate pyruvate lyase enzymes. Salicylic acid glucosides (SAG and SGE) are produced by glucosyltransferases (SAGT); while, methylation of SA is performed by the methyltransferases enzyme. The chemical structures and its subcellular localization of SA substrates and derivatives are shown.

Salicylic acid is probably present in all plants, however, its concentration can fluctuate widely between the species and even between members of the same family (Raskin et al., 1990). For example, tobacco leaves can contain <100 ng/g fresh weight (FW) of SA, while in potato can reach 10 μg/g FW (Malamy et al., 1992; Coquoz et al., 1998). In the model plant *Arabidopsis thaliana*, the basal level of SA has been quantified between 0.250 and 1 μg/g FW (Nawrath and Métraux, 1999; Wildermuth et al., 2001). Several reviews have recently made a detailed description of SA biosynthesis (Dempsey et al., 2011; Dempsey and Klessig, 2017; Klessig et al., 2018). To date, the biosynthesis of SA has not been fully defined, however, genetic and biochemical evidence pointed out that SA can be produced from two independent and compartmentalized pathways: the isochorismate (IC) pathway localized into the plastids and the phenylalanine ammonia-lyase (PAL) pathway that takes place in the cytosol (Figure 2). Both pathways begin by the accumulation of chorismic acid, produced by shikimic acid biosynthesis. In the IC pathway, chorismic acid is converted into isochorismic acid (IC) by isochorismate synthase and then through the activity of isochorismate pyruvate lyase (IPL) SA is produced in the chloroplast and afterward exported to the cytosol (Fragnière et al., 2011). On the other hand, in the PAL pathway, prephenic acid is produced from the chorismic acid by chorismate mutase (CM), then reduced to L-phenylalanine (Phe) that is converted into *trans*-cinnamic acid (*t*-CA) by PAL activity. From *t*-CA two metabolites can be produced: *ortho*-courmaric acid (*o*-CA) and benzaldehyde. SA can be synthesized directly from *o*-CA, while benzaldehyde is first transformed into benzoic acid (BA) by aldehyde oxidase (AAO) and then to SA by the activity of benzoic acid 2-hydroxylase (BA2H) (Figure 2). Once SA is synthesized, its levels are regulated by a number of chemical modifications, to produce inactive forms, including salicyloyl glucose ester (SGE), SA O-β-glucoside (SAG), methyl salicylate (MeSA), and methyl salicylate O-β-glucoside (MeSAG). These inactive molecules can be stored until required to activate the SA-triggered responses.

In *Arabidopsis thaliana*, glucosylation of SA is performed by the action of UDP-glucosyltransferase enzymes UGT74F1 and UGT74F2 (Lim et al., 2002; Dean and Delaney, 2008). Both genes *UGT74F1* and *UGT74F2* are SA-induced and localized in the cytosol (Dean and Delaney, 2008; Park et al., 2017). The *ugt74f1* mutant accumulates less SAG than *ugt74f2* mutant and wild-type plants, while SGE was not formed in the *ugt74f2* background (Dean and Delaney, 2008). It has been demonstrated that UGT74F1 catalyzes the formation of SAG and UGT74F2 forms primarily SGE, but also synthetizes SAG (Dean and Delaney, 2008; Dempsey et al., 2011). On the other hand, SA produces MeSA by the action of a carboxyl methyltransferase (SAMT) (Ross et al., 1999; Zubieta et al., 2003). In *Arabidopsis thaliana*, *AtBSMT1* encodes for a carboxyl methyltransferase, which may use either BA or SA as substrates to form MeSA (Chen et al., 2003) (Figure 2). It was observed that *AtBSMT1* gene expression and hence MeSA production were induced in *Arabidopsis thaliana* leaves by alamethicin treatment, a pore-forming peptide that emulates pathogen damage (Chen et al., 2003). Therefore, it is suggested that AtBSMT1 performs MeSA production mainly during pathogen infection (Liu et al., 2009). Mutants impaired in the expression of *AtBSMT1* did not accumulate MeSA after pathogen attack, while overexpression of *AtBSMT1* led to an incremented accumulation of MeSA at the infection zone (Liu et al., 2009). Interestingly, overexpression and mutants of *AtBSMT1* were not able to accumulate SA or SAG at the distal leaves and they did not establish SAR (Liu et al., 2009). Likely, the overexpression of *AtBSMT1* leads to excessive conversion of free SA to MeSA, which avoids the required accumulation of SA to develop SAR at the systemic leaves. A similar observation was made when *Arabidopsis thaliana* plants overexpress the *OsBSMT1* gene from rice, which accumulated MeSA constitutively and failed to accumulate SA or SAG and were vulnerable to pathogen disease (Koo et al., 2007). Surprisingly, the increase in MeSA levels released by transgenic plants was enough to provoke SAR in nearby wild-type plants in an ICS1-independent process (Koo et al., 2007). Finally, in *Nicotiana benthamiana* virus-infected plants, SAR was impaired when the *SAMT* gene was silenced (Zhu et al., 2014).

Taken together, the fact that SA is produced into the chloroplast and in the cytosol, its inactive form (SAG) is stored in different cellular compartments (Figure 2) and afterward spread all over the plant to participate in SAR, suggests an intra and extracellular movement, that should be coordinated by the action of transporters.

## The Journey of SA

**FIGURE 3:**
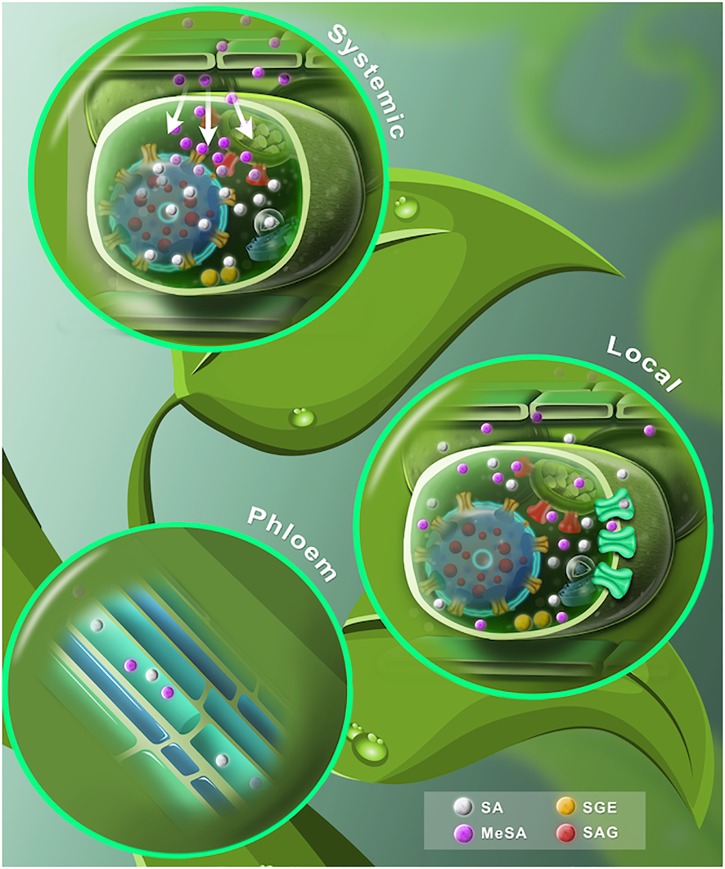
A proposed dynamic model of free and conjugated SA. **(Lower leaf)** The SA synthesized by ICS and PAL pathways is shown in gray spheres. At the chloroplast, SA is translocated to the cytosol through EDS5 transporters (red carrier). Once extruded, SA is conjugated with glucose to form SAG or SGE (red and yellow spheres, respectively). Later, SAG is transported into the vacuole by ABC transporter/H+ -antiporter systems (yellow carriers). SA may spread out to the apoplast by a carrier-mediated system (green carriers). SA is converted to volatile MeSA (purple spheres) by carboxyl methyltransferase enzyme (SAMT). **(Stem)** SA phloem transport may be based on a symplastic outer cell transport, phloem apoplast intake through an ion trap mechanism and an apoplast intake mediated by a carrier system. MeSA also can be found in the phloem. **(Upper leaf)** After a pathogen attack, SA levels rise in the primary infected tissue. SA is converted to MeSA (purple spheres). The accumulating MeSA is translocated to the uninoculated systemic tissue. MeSA is demethylated to form SA and induces *de-novo* synthesis of SA at the distal tissue.

Similarly, to animal hormones, phytohormones are frequently synthesized in a different place to where they develop their function, thus requiring either local or long-distance communication. Plants employ numerous delivery mechanisms that depend on the distance and direction of the transport (Bonnemain et al., 2013; Park et al., 2017). In the next sections, we examine the most exciting advances about intracellular, cell-to-cell, and long-distance transport of SA (Table 1 and Figure 3).

**Table 1:** List of plant SA transporter mechanisms.

Plant species	Carrier name	Transporter class	Substrate	Subcellular localization	References
*Arabidopsis thaliana*	EDS5	Multidrug and toxin extrusion-like (MATE)	SA	Chloroplast envelope	(Serrano et al., 2013)
*Glycine max*	n.d.	ATP-binding cassette (ABC)	SAG	Vacuole tonoplast	(Dean and Mills, 2004)
*Beta vulgaris*	n.d.	H^+^-antiporter^∗^	SAG	Vacuole tonoplast	(Dean and Mills, 2004)
*Nicotiana tabacum*	n.d.	H^+^-antiporter^∗^	SAG	Vacuole tonoplast	(Dean et al., 2005)
*A. thaliana*	n.d.	ABC/H^+^-antiporter^∗^	SAG	Vacuole tonoplast	(Vaca et al., 2017)
*Ricinus communis*	n.d.	pH-dependent carrier	SA	Phloem	(Rocher et al., 2006, 2009)
*N. tabacum*		Diffusion	MeSA	Phloem	(Liu et al., 2011)
*A. thaliana*		Diffusion	MeSA	Phloem	(Liu et al., 2011)


*n.d., not determined. ^∗^likely MATE.*

## Intracellular Transport: From SA Synthesis to Storage

The synthesis of SA, induced by biotic and abiotic stress, is localized in the chloroplast and afterward transported to the cytosol (Dean et al., 2005; Garcion et al., 2008; Fragnière et al., 2011). However, the molecular mechanism involved in this active transport was for a long time unknown. At the beginning of 2000’s, two *Arabidopsis thaliana* mutants, named SA induction–deficient (*sid1* and *sid2*) were identified (Nawrath and Métraux, 1999). *sid* mutants were described to be impaired in SA biosynthesis and to show enhanced disease susceptibility to bacterial and fungal pathogens (Nawrath and Métraux, 1999). *sid1* was identified to be allelic to *ENHANCED DISEASE SUSCEPTIBILITY 5* (*EDS5*), a member of the multidrug and toxin extrusion (MATE) transporter family (Nawrath and Métraux, 1999; Nawrath et al., 2002). *Arabidopsis thaliana* plants overexpressing *EDS5* show enhanced resistance to viruses, validating the role of *EDS5* during the plant defense responses (Ishihara et al., 2008). Additionally, other reports have shown that EDS5 is localized at the chloroplast envelope membrane, suggesting that it might be involved in SA transport (Serrano et al., 2013; Yamasaki et al., 2013). Analyzing the movement of radiolabeled SA in isolated chloroplasts overexpressing *EDS5* from *Arabidopsis thaliana*, and in a yeast heterologous system, it was confirmed that EDS5 is involved in the export of SA from the chloroplast to the cytosol (Serrano et al., 2013).

Interestingly, the *EDS5* homolog (*EDS5H*) also shows a chloroplastic envelope location, but, however, is induced neither by pathogens nor by application of exogenous SA and its mutant *eds5h* is not impaired in the biosynthesis of SA (Parinthawong et al., 2015). EDS5H most likely transports other phenolic compounds, but not SA, which suggests that the protein-mediated transport system of SA is only performed by specific carriers (Parinthawong et al., 2015). This evidence suggests that EDS5, to our knowledge, is the unique SA plant transporter identified so far involved in its movement from chloroplast to cytosol.

Once synthesized or transported in the cytosol, SA can undergo conjugation with glucose in order to render an inactive form that is water soluble for storage (Figure 3). Depending on where glucose is attached, either the hydroxyl group or the carboxyl group, SA can be transformed into SA-glucoside (SAG) or SA glucose ester (SGE), respectively (Lim et al., 2002; Dean et al., 2003, 2005; Dean and Mills, 2004; Park et al., 2007). It is known that glucosides, such as flavonoid glucosides and SAG, are stable forms for storage of other small phenolic compounds, while glucose esters, such as SGE, may act as intermediary substrates in a biosynthetic pathway (Petrussa et al., 2013; Vaca et al., 2017). Interestingly, reports in tobacco and poplar suggest that the glucose ester of BA and SGE might be precursors for SA formation, indicating that SGE might accumulate in the cytosol to be used as a ready-to-use precursor for SA while SAG is accumulated in the vacuole until the plant needs it (Chong et al., 2001; Ruuhola and Julkunen-Tiitto, 2003).

It has been shown that SAG is transported into the vacuole in various plant species, such as soybean, tobacco, red beet and *Arabidopsis thaliana* (Table 1) (Dean and Mills, 2004; Dean et al., 2005; Vaca et al., 2017). For soybean, SAG appears to be exported into the vacuole by ATP-binding cassette (ABC) transporters (Dean and Mills, 2004). In tobacco and red beet, transport is carried out by H^+^-antiporters (Dean et al., 2005). While in *Arabidopsis thaliana*, SAG is stored in the vacuole and SGE is only found in the cytoplasm (Vaca et al., 2017). An uptake analysis of radiolabeled SAG, in vacuoles of *Arabidopsis thaliana*, revealed that it is translocated toward the vacuole through a MgATP-dependent process (Vaca et al., 2017). Moreover, vacuolar transport of SAG was blocked by inhibitors of ABC transporters and H^+^-antiporter systems, indicating that glycosylated SA transport may be performed by these types of pumps (Vaca et al., 2017).

SA-glucoside is reconverted to the active form of SA when hydrolyzed. In tobacco, SAG is reconverted to SA at the apoplast (Hennig et al., 1993), which makes sense with the extracellular localization of β-glucosidases in dicotyledonous plants and also corresponds to the role played by these enzymes during plant pathogen interactions (Morant et al., 2008). However, it is still unknown if vacuolar SAG is transported to the apoplast and/or if it is released after a pathogen-induced response. Additionally, it cannot be discounted that intracellular enzymes may hydrolyze SAG under special conditions (Dempsey et al., 2011). Taking together, these observations indicate that the intracellular level of SA and its inactive forms are under the control of transporters.

## Cell to Cell Transport: Influx and Efflux of SA

Once SA is synthesized inside the cells, the next step in the SA journey is its dissemination to neighboring cells (Figure 3) (Kawano et al., 2004). Often SA is spread via the apoplast (Lim et al., 2016; Singh et al., 2017). Because of its chemical features, such as weak acid and poor water solubility, SA crosses through animal and plant cell plasma membranes by pH-dependent diffusion and carrier-mediated mechanisms (Chatton and Roch-Ramel, 1992; Takanaga et al., 1994; Chen et al., 2001; Emoto et al., 2002; Bonnemain et al., 2013). In mammals, a monocarboxylate transporter is localized at the cell plasma membrane (Enerson and Drewes, 2003). In plants, the influx of radiolabeled SA has been reported in the aquatic plant *Lemna gibba*, where around 90% of 10 μM SA applied to the medium was taken up in a half hour, however, the mechanisms by which the SA was internalized are unknown (Ben-Tal and Cleland, 1982). Nevertheless, after uptake, SA was localized either in the cytosol or vacuole and no further plasmodesmata transport was observed in *L. gibba* cells (Ben-Tal and Cleland, 1982). It is likely that SA was quickly conjugated with glucose and stored in vacuoles.

Interestingly, SA uptake was faster in a tobacco cell suspension than in *L. gibba* plants, taking up to 200 μM SA in just 5 min (Chen et al., 2001). Nevertheless, after 5 h, over 90% of the radiolabeled SA absorbed was released to the medium. The SA influx could be inhibited by adding a chelating agent (EGTA), but the efflux was restored by addition of Ca^2+^ and moreover, a protein synthesis inhibitor blocked SA excretion (Chen et al., 2001; Kawano et al., 2004). The authors suggest the presence of a SA efflux transporter that may be induced at high SA concentrations and which may involve ROS, Ca^2+^, *de-novo* protein synthesis and a protein phosphorylation signaling pathway (Chen et al., 2001; Kawano et al., 2004; Bonnemain et al., 2013). However, a constitutive SA efflux carrier involved during low SA concentrations, independent of ROS, Ca2^+^, and protein kinase cascade signaling has been also proposed (Kawano et al., 2004; Bonnemain et al., 2013).

## Long-Distance Transport

Along with azelaic acid (AzA), glycerol-3-phosphate (G3P), methyl jasmonate (MeJA) and pipecolic acid (Pip), SA participates as a critical long-distance inducer for SAR (Conrath et al., 2015; Klessig et al., 2018). Rapid translocation of radiolabeled SA, injected to the end of tobacco leaf petioles, was observed in systemic neighboring upper and lower leaves (Ohashi et al., 2004). When ^14^C-labeled BA was applied to cucumber cotyledons infected with tobacco necrosis virus, labeled SA was subsequently found in the phloem and in the upper uninfected leaf (Mölders et al., 1996). During pathogen infection, SA is accumulated in the phloem, via the apoplast in *Arabidopsis thaliana* and tobacco plants (Yalpani et al., 1991; Zhu et al., 2014; Lim et al., 2016). Additionally, it has been reported that translocation of radiolabeled SA occurs from tobacco virus-inoculated leaves toward uninfected distal zones (Shulaev et al., 1995). SA translocation by the xylem has also been reported in tobacco and *Ricinus communis* seedlings, although the contribution of xylem in SA-mediated transport to develop SAR is not yet clear (Ohashi et al., 2004; Rocher et al., 2006).

In *Ricinus communis* seedlings, the SA phloem transport mechanisms comprise a high specificity pH-dependent carrier system, which may be placed in internal tissues, mainly at the cotyledon veins (Table 1) (Rocher et al., 2006, 2009). The authors suggest that SA phloem loading may be based on a symplastic outer cell transport, phloem apoplast intake, through an ion trap mechanism and an apoplast intake mediated by a carrier system (Figure 3) (Rocher et al., 2009).

The major barrier to prevent the free diffusion of SA is the plant cuticle (Niederl et al., 1998; Ohashi et al., 2004). Controversially, it is well known that the exogenous application of SA, either by seed priming (soaked seeds before sowing), the addition of SA to a hydroponic solution or spraying plants with SA solution, is favorable for plant growth and helps to protect them against abiotic stresses (Hayat et al., 2010). For instance, seed priming with SA, increased the activities of antioxidant enzymes in young pea (*Pisum sativum* L.) and leads to the *de-novo* synthesis of SA (Szalai et al., 2011). When sweet basil (*Ocimum baslicum* L.) plants were sprayed with citric and SA, supplemented with a surfactant agent, the root nutrient acquisition pattern changed to assimilating more boron and sulfur, therefore enhancing their uptake to plant shoot, thus increasing fresh biomass and photosynthetic efficiency (Ghazijahani et al., 2014). Moreover, in wheat plants (*Triticum aestivum*) the abiotic stress caused by the insecticide chlorpyrifos was mitigated by spraying exogenous SA; probably due to an activity improvement of antioxidant enzymes. Also, SA was able to avoid the uptake of chlorpyrifos in wheat plants (Wang and Zhang, 2017). However, exogenous applications of SA are often carried out to run-off, with the possibility that could enter via stomata and that soft mechanical damage can induce the defense mechanisms (Benikhlef et al., 2013). Nevertheless, the mechanisms involved in the assimilation and/or transport of SA under these conditions has not been described.

It has been demonstrated that cuticle is hardly permeable to SA, unless it is converted to a volatile form (Figure 3). MeSA is a volatile SA derivative and it acts as an airborne defense signal (Table 1) (Koo et al., 2007; Park et al., 2007). MeSA is mobilized through the phloem to activate SAR (Yalpani et al., 1991; Kumar and Klessig, 2003). MeSA becomes active when it is reversed to SA by the MeSA esterase activity of SA-binding protein 2 (SABP2) in the systemic tissue (Figure 2) (Seskar et al., 1998; Park et al., 2007, 2009; Vlot et al., 2008; Manosalva et al., 2010). In fact, mutations in either *SAMT* or *SABP2*, compromise SAR (Vlot et al., 2008). Altogether, this evidence supports the knowledge that the transport of SA and MeSA serves as a long-distance phloem-mobile signal and that correct homeostasis must be carried out for a successful triggering of SAR. While SA belongs to an orchestrated mechanism that induces SAR, it is not the only one – but it is a fundamental player in the activation of such a significant task of plants when facing the attack of pathogens.

## What to Expect About SA Transport in the Future?

Numerous studies confirm that SA plays a crucial role during plant growth and development and in particular during the plant innate immunity (Klessig et al., 2018). In contrast, discovery and characterization of transport systems and signaling pathways remain largely elusive. While genomic approaches, radiolabeled SA tracking analysis and inhibition of channel gating, have provided insights in SA intracellular and long-distance translocation (Dean and Mills, 2004; Park et al., 2007; Serrano et al., 2013; Zhu et al., 2014; Vaca et al., 2017), there are too many open questions to be answered about the molecular and cellular mechanisms that guide the trafficking of intra- and extra-cellular SA.

We now need to move beyond, to approaches that allow identifying new SA carriers, receptors and targets. For example, the mapping of SA-mediated protein-protein interactions can be a suitable tool for this aim. It is likely that more MATE- and ABC-type transporters will be found to be involved in the transport of SA, since these transporter families have been reported to transport such a diversity of chemicals, including other phytohormones (Zhang et al., 2014; Hwang et al., 2016). Membrane protein-protein interaction and transmission surface plasmon resonance (TSPR) approaches among others, might offer tremendous insight in new transport and receptors of SA (Lertvachirapaiboon et al., 2018). For instance, in the future we shall be capable of monitoring the distribution of intracellular SA pools by biosensor imaging development, using these newly identified SA receptors (Jones, 2016).

Additionally, mathematical modeling has been used to predict phytohormone transport and signaling pathways (Voß et al., 2014) e.g., the case of the cellular and long-distance transport of auxin in tobacco and *Arabidopsis thaliana*, respectively (Hošek et al., 2012; Boot et al., 2016). Also, to predict sequential induction of both auxin efflux and influx carriers in the regulation of lateral root emergence in *Arabidopsis thaliana* (Péret et al., 2013). Modeling predictions are useful to select appropriate experimental systems and generate hypothetical models of SA transport (Otto et al., 2018), which in parallel with experimental observations will be a powerful tool to develop and establish models of the regulation mechanism for SA transport.

## Author Contributions

IM-L, NYA-B, AB, and MS wrote and revised the manuscript. All the authors approved the final version of the manuscript.

## Conflict of Interest Statement

The authors declare that the research was conducted in the absence of any commercial or financial relationships that could be construed as a potential conflict of interest.
